# Supplementary material for: The Role of Physical Activity in Adjunctive Nursing Management of Neuro-Degenerative Diseases among Older Adults: A Systematic Review of Interventional Studies
Source: Life (Basel). 2024 May 7;14(5):597. doi: 10.3390/life14050597 (PMC11122640; doi:10.3390/life14050597)
Supplement: Supplementary file 1 [file life-14-00597-s001.zip › life-2967683-supplementary.pdf]

SUPPLEMNATRY Table S1. The extraction table of the included studies

Author and Year	Study Design	Sample Size	Type of Neurodegenerative Disease	Type of Physical Activity Intervention	Duration of Intervention	Outcome Measures	Key Findings
Rivas-Campo et al., 2023[47]	Single-Blind Randomized Controlled Trial	132 (64 intervention, 68 control)	Mild Cognitive Impairment (MCI)	High-Intensity Intervallic Functional Training (HIFT)	3 months	Montreal Cognitive Assessment (MoCA), Trail Making Test (TMT) A & B, Verbal Fluency Animal Test (VFAT), Digit Symbol Substitution Test (DSST), D2 Test	The intervention group showed significant improvements in cognitive functions, attention, and verbal fluency compared to the control group. No significant results for selective attention or processing speed.
Baker et al., 2010 [48]	Six-month randomized controlled clinical trial	33 (17 women, 16 men)	Amnestic Mild Cognitive Impairment (MCI)	High-intensity aerobic exercise vs. stretching control group	6 months	Performance on Symbol-Digit Modalities, Verbal Fluency, Stroop, Trails B, Task Switching, Story Recall, and List Learning. Fasting plasma levels of insulin, cortisol, brain-derived neurotrophic factor, insulin-like growth factor-I, and β-amyloids 40 and 42.	Aerobic exercise improved cognitive function, especially in executive control processes, and had sex-specific effects on glucose metabolism and hypothalamic-pituitary-adrenal axis and trophic activity. Improvements were more pronounced for women in terms of executive function

							and metabolic responses.
Rivas-Campo et al., 2023[58]	Blind Randomized Controlled Clinical Trial	169	Mild Cognitive Impairment (MCI)	High-Intensity Functional Training (HIFT)	12 weeks	Gait stability, balance, independence in activities of daily living, functional capacity, frailty	Significant improvements in gait stability, balance, and independence in activities of daily living in the intervention group compared to the control group. No significant changes in frailty classification.
Pallesen et al., 2019[59]	Pilot Randomised Controlled Trial	30	Post-stroke Cognitive Impairments	High-Intensity vs. Low-Intensity Aerobic Exercise	4 weeks	Trail Making Test B, neuropsychological tests assessing various cognitive functions	The high-intensity group showed significant improvements in Trail Making Test B, indicating enhancements in processing speed and divided attention, compared to the low-intensity group. However, the improvements in cognitive functions were not conclusively attributed to the

							intervention due to potential ceiling effects and baseline inequalities.
Vieira de Moraes Filho et al., 2020[60]	Randomized Controlled Trial	40 (25 intervention, 15 control)	Parkinson's Disease (PD)	Progressive Resistance Training (PRT)	9 weeks	Bradykinesia UPDRS subscale, knee extensors isokinetic strength, Ten Meters Walk Test, Timed Up&Go Test, 30-Second Chair Stand	PRT significantly reduced bradykinesia and improved functional performance in PD patients without significant changes in muscle strength, suggesting neural enhancements.
Smaili et al., 2018[61]	Randomized Clinical Trial	40	Parkinson's Disease	Neurofunctional Training vs. Resistance Training	12 weeks (24 sessions)	Gait parameters (stride length, step length, number of steps, gait speed, cadence), Quality of Life (PDQL, PDQ-39)	Neurofunctional training showed significant improvements in all gait parameters and quality of life compared to resistance training, emphasizing the superiority of neurofunctional approaches for this patient population.
Tyndall et al., 2013[62]	Combined Quasi-Experimental and Prospective Cohort Study	250	General Aging (Older Adults)	Aerobic Exercise	6 Months	Cerebrovascular Function, Cognitive Function, Cardiovascular Fitness, Mood and Sleep Assessments	The study aimed to assess the impact of a structured 6-month aerobic exercise program on cerebrovascular

							function and cognitive abilities in older individuals, and to determine the extent of any changes post-intervention.
Sungkarat et al., 2016[63]	Randomized Controlled Trial	66	Amnestic Mild Cognitive Impairment (a-MCI)	Tai Chi (combined center- and home-based training)	3 weeks center-based, 12 weeks home-based	Cognitive tests (Logical Memory, Block Design, Digit Span, Trail-Making Test B–A), Physiological Profile Assessment (PPA) for fall risk	Tai Chi significantly improved cognitive function (memory, visuospatial ability, executive function) and reduced physiological fall risk compared to control group, demonstrating its potential benefit for older adults with a-MCI.
Chen et al., 2023[64]	Randomized Clinical Trial	328	Mild Cognitive Impairment (MCI) with Type 2 Diabetes	Tai Chi Chuan vs. Fitness Walking	24 weeks	Montreal Cognitive Assessment (MoCA), other cognitive subdomain measures, and blood metabolic indices	Tai Chi Chuan showed significantly more benefit on global cognitive function at 36 weeks compared with fitness walking, with no significant difference at 24 weeks.

Liu et al., 2022[65]	Randomized Control Trial	50	Mild Cognitive Impairment (MCI)	Exergaming-Based Tai Chi (EXER-TC) vs. Traditional Tai Chi (TC) vs. Control	12 weeks	Montreal Cognitive Assessment (MoCA), Trail Making Test (TMT) Parts A and B, Stroop Color and Word Test (SCWT), Gait Speed, Dual-Task Cost (DTC)	Both EXER-TC and TC groups showed improvements in cognitive function tests and dual-task gait performance compared to the control group, with no significant differences between EXER-TC and TC groups.
Carapellotti et al., 2022[49]	Pilot and Feasibility Study	10	Parkinson's Disease	Dance (Dance for PD® model)	12 weeks	MDS-UPDRS III, TUG, DT-TUG, Sensory Organization Test, MoCA, Trail Making Tests A&B, Digit Symbol Substitution Test, Digit Span, PDQ-39, FOG-Q, PHQ-9, FES-I	The dance program was feasible and well-received, showing improvements in functional mobility, symptoms of depression, and bodily discomfort without adverse effects. The study supports dance's potential for physical, emotional, mental, and social health benefits in Parkinson's patients.
Morris et al., 2023[50]	Clinical Trial Process Evaluation	12 PD participants, 4 dance instructors, 2 physiotherapists	Parkinson's Disease	Online Dance Program (ParkinDANCE Online)	4 weeks	Not specified in the visible content	The study demonstrated the feasibility of delivering an online

							dance program to individuals with early PD, highlighting engagement and practicality without reported adverse events.
Koo, Jang, and Kwon, 2021[51]	Randomized Controlled Trial	24	Mild Dementia	Dual-Task Training (DTT) vs. Single-Task Training (STT)	8 weeks	Stride length, stride velocity, cadence, step length, swing phase, stance phase, double support phase	DTT led to significant improvements in gait parameters compared to STT, suggesting its effectiveness in improving gait function in elderly patients with mild dementia.
Maeneja et al., 2023[52]	Randomized Clinical Trial	34	Stroke	Aerobic Physical Exercise vs. Dual-Task Cognitive Walking	12 weeks	Mini-Mental State Examination (MMSE), d2 Test of Attention	Aerobic physical exercise showed more significant improvements in cognitive attention scores and decreased cognitive impairment and perception of exertion compared to the dual-task group.

Kim et al., 2016[53]	6-Month Randomized Controlled Trial	33	Alzheimer's Disease (moderate to severe)	Physical Exercise with Multicomponent Cognitive Program (MCP) vs. MCP alone	6 months	ADAS-cog, MMSE, CDT, physical performance measures	The intervention group showed no significant cognitive improvement over the control group in primary analysis. However, secondary analyses adjusted for baseline values, age, sex, and education showed significant improvements in ADAS-cog scores. Physical performance improved significantly in the intervention group.
Li et al., 2021[54]	Randomized Controlled Trial	90 (84 completed)	Mild Cognitive Impairment (MCI)	Multi-component exercise training	6 months	CM-PPT, MMSE, MoCA	Multi-component exercise training significantly improved both physical and cognitive functions in elderly participants with MCI, demonstrating its effectiveness as a non-drug intervention for this population.

Chow et al., 2022[55]	Cluster Randomized Controlled Trial	285	General Aging (Older Adults)	Various combinations of cognitive and/or physical training	4 months	Cognitive and physical fitness measures	The study aims to determine the effects of different combinations of cognitive and physical training on cognition and physical fitness of older adults, comparing the efficacy of various training modes.
Pompeu et al., 2012[56]	Single-Blind Randomized Clinical Trial	32	Parkinson’s Disease	Nintendo Wii-based Motor and Cognitive Training	7 weeks	UPDRS-II, balance, cognitive performance	Both the experimental (Wii-based training) and control groups (balance exercise therapy) improved in UPDRS-II scores, with no significant difference between groups, indicating Wii-based training and conventional balance exercises similarly improved activities of daily living in Parkinson's disease patients.
Hariprasad et al., 2013[57]	Randomized Controlled Study	120 (62 yoga group, 58 waitlist control)	General Cognitive Decline in the Elderly	Yoga-Based Intervention	6 months	RAVLT, CFT, WMS-digit and spatial span, COWA test, Stroop Color Word Interference	The yoga group showed significant improvements in immediate and

			Test, Trail Making Test A and B	delayed recall of verbal and visual memory, attention and working memory (WMS-spatial span), verbal fluency (COWA), executive function (Stroop interference), and processing speed (Trail Making Test-A) compared to the waitlist group.
